# The Adverse Impact of Pregestational Prediabetes Contributes to HELLP Syndrome Development

**DOI:** 10.3390/biology14121707

**Published:** 2025-11-30

**Authors:** Anelisiwe Siboto, Asiphaphola Ludidi, Nombuso Xulu, Ayanda Nkosi, Ntethelelo Sibiya, Andile Khathi, Phikelelani Siphosethu Ngubane

**Affiliations:** 1 School of Laboratory Medicine and Medical Sciences, University of KwaZulu Natal, Durban 4001, South Africa; ludidia@ukzn.ac.za (A.L.); 215019278@stu.ukzn.ac.za (N.X.); 216000895@stu.ukzn.ac.za (A.N.); khathia@ukzn.ac.za (A.K.); ngubanep1@ukzn.ac.za (P.S.N.); 2 Division of Pharmacology, Faculty of Pharmacy, Rhodes University, Makhanda 6139, South Africa; n.sibiya@ru.ac.za

**Keywords:** HELLP, pregestational PD, liver function

## Abstract

Introduction: Both preeclampsia (PE) and prediabetes (PD) are known hypertensive disorders of pregnancy, and a correlation has been shown between these two diseases. A recent study in our laboratory has shown that pregestational PD is a risk factor for developing PE during pregnancy, as pregestational PD increased antiangiogenic factors. However, pregestational PD antiangiogenic release has not been shown to be associated with liver dysfunction. Therefore, this study seeks to investigate pregestational PD as a risk factor for hemolysis, elevated liver enzymes, and low platelet (HELLP) syndrome. Materials and Methods: Animals were divided into a normal pregnant group (ND), a preeclamptic pregnant group (PE), and a prediabetic pregnant group (PD). On gestational day (GND) 19, animals were sacrificed, and blood and liver tissues were collected to measure antioxidant protection and lipid peroxidation parameters, liver TGs, liver enzymes, TNF-α, IL-6, and hematology parameters. Results: The results showed significant increases in liver TGs, liver enzymes, TNF-α, IL-6, and hematology parameters in the PE and PD pregnant groups compared to the ND group. Conclusions: These findings suggest that pregestational PD predisposes patients to metabolic and inflammatory changes associated with HELLP syndrome. To our knowledge, this is the first study to demonstrate a link between pregestational PD and HELLP syndrome-related complications in a preclinical model, highlighting the importance of monitoring metabolic health before pregnancy.

## Simple Summary

Previous studies have shown that having prediabetes before pregnancy is a risk factor for preeclampsia, causing harmful effects on the placenta and harming the baby. We were interested in finding out whether these harmful effects can also affect other organs of the mother. This study examined whether having prediabetes before pregnancy increases the risk of hemolysis, elevated liver enzymes, and low platelet syndrome, a serious pregnancy complication that is characterized by liver dysfunction and abnormalities in blood parameters. We used animal models, and we compared normal, preeclamptic, and prediabetic pregnancies. We found higher levels of liver fats, liver enzymes, inflammatory markers, and blood abnormalities in both the preeclamptic and prediabetic pregnant groups compared to normal pregnancies. The results suggest that pregestational prediabetes may trigger metabolic and inflammatory changes that can lead to hemolysis, elevated liver enzymes, and low platelet syndrome, highlighting the need to monitor women’s metabolic health before pregnancy.

## 1. Introduction

Studies have shown that preeclampsia (PE) complicates about 2–4% of pregnancies globally [1], whereas there is limited data on the prevalence of pregnancy complicated by prediabetes (PD). PE and PD are associated with hypertensive disorders during pregnancy and have been shown to correlate with one another [2,3]. Additionally, both pathologies have adverse effects on both the mother and fetus, such as a significantly increased risk of developing hypertension, heart failure, end-stage chronic kidney disease (CKD), and type 2 diabetes mellitus (T2DM) [4,5]. The primary mechanism involved in the etiology of PE is inadequate trophoblast invasion of maternal spiral arteries. This leads to maternal global endothelial dysfunction and hypoxic conditions, driven by antiangiogenic factors produced by a stressed placenta [6]. A recent study by Ludidi et al. has shown that PD is a risk factor for developing PE during pregnancy, as PD is associated with an increase in antiangiogenic factors [3]. The upregulation of antiangiogenic factors in PE has been shown to exert its adverse effects on the maternal endothelium, including all areas of fenestrated endothelium in the liver, thus causing hepatocytes to become leaky and release liver enzymes into the maternal circulation [7].

Severe preeclampsia can cause HELLP syndrome, which is marked by hemolysis, high liver enzyme levels, and low platelet counts [8]. HELLP syndrome has been shown to impact the liver’s synthetic function, potentially affecting albumin production [9]. Similar metabolic and hepatic abnormalities have been observed in prediabetic male Sprague-Dawley rats [10]. However, these abnormalities have not been shown to occur in prediabetic pregnant female Sprague-Dawley rats.

Therefore, the present study aimed to investigate whether pregestational PD represents a risk factor for the development of HELLP syndrome. Specifically, we sought to determine its contribution to PE-associated liver dysfunction by assessing liver triglyceride (TG) accumulation, alterations in liver enzyme activity, elevations in inflammatory markers (TNF-α and IL-6), and changes in hematological parameters.

## 2. Materials and Methods

### 2.1. Drugs and Chemicals

Drugs were sourced from standard pharmaceutical suppliers. All other chemicals were of analytical grade and were purchased from standard commercial suppliers.

### 2.2. Animals

This study was approved by the University of Kwazulu-Natal ethics committee as the procedures involving animals and their care were conducted in conformity with the institutional guidelines. Approved ethics number: AREC/025/19D. First, 3-week-old female Sprague-Dawley rats (n = 18) bred and housed in the Biomedical Research Unit of the University of Kwazulu-Natal were used in the present study. The animals were maintained under standard housing conditions of constant temperature (22 ± 2 °C), a CO_2_ content of ˂5000 pm, a relative humidity of 55 ± 5%, illumination (12 h light/dark cycles), noise levels of less than 65 decibels, and ad libitum access to standard rat chow (29% protein, 55% carbohydrate, and 16% fat) and water.

#### Experimental Design

**Figure 1:**
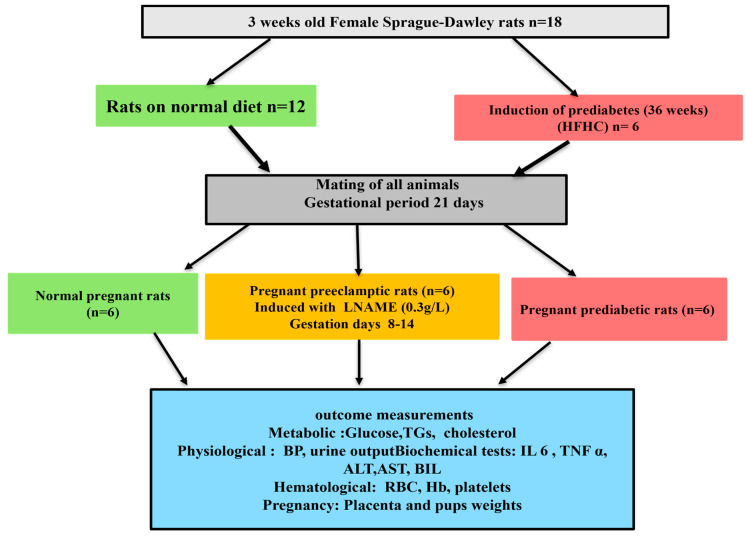
Experimental protocol.

The role of pregestational PD as a risk factor for PE was studied in the following groups. Group 1: normal pregnant control rats (ND) that received normal standard chow and normal drinking water throughout this study (n = 6/group); Group 2: PD pregnant rats that received a high-carbohydrate, high-fat (HCHF) diet + 15% fructose throughout this study (n = 6/group); Group 3: PE pregnant control rats that received normal standard chow and 0.3 g/l N(ω)-nitro-L-arginine methyl ester (LNAME) in drinking water from gestational day (GND) 8 to GND 14 (n = 6/group). On GND 0, 9, and 18, non-fasting glucose and body weight were measured (Figure 1).

### 2.3. Induction of PD

After 1 week of acclimatization, PD was induced in 4-week-old female Sprague-Dawley rats (n = 6) using an established protocol from our laboratory with slight modification [11,12]. Briefly, the experimental animals were exposed to a high-carbohydrate, high-fat diet and 15% fructose (HCHF diet + 15% fructose) in drinking water for 36 weeks [11]. The control group (n = 6) and preeclamptic group (n = 6) received standard chow diet and normal water for 36 weeks. During the induction period, all the rats were also acclimatized to a blood pressure machine once a month.

#### 2.3.1. Criteria for PD

The American Diabetes Federation criteria were used to diagnose PD in all animals. Animals that have prediabetic symptoms including a fasting blood glucose concentration of 6.1–7.1 mmol/L, a two-hour glucose concentration of 7.1–8.1 mmol/L in the oral glucose tolerance test, and a plasma triglyceride concentration of greater than 2 mmol/L were considered prediabetic. Blood samples were collected from the tail vein through the tail pricking method. Glucose was determined using an Elite^®^ glucometer (Ascensia Diabetes Care Bangladesh, Basel, Switzerland), and blood TGs were determined using an AccuTrend Plus cholesterol meter (Roche Diagnostics, Johannesburg, South Africa).

#### 2.3.2. Experimental Diet

The composition of a high-fat, high-carbohydrate diet was customized as shown in Table 1. The experimental diet formula is made up of the following: carbohydrate (55% Kcal/g), fats (30% Kcal/g), and proteins (15% Kcal/g). The experimental animals’ drinking water was supplemented with 15% fructose.

#### 2.3.3. Diet Composition

The control group received standard rat chow composed of approximately 29% protein, 55% carbohydrate, and 16% fat. The main sources of protein were soybean meal and casein, while carbohydrates were derived primarily from maize starch and wheat by-products. The fat content of the chow was supplied mainly by plant oils.

To induce prediabetes, animals were fed a high-fat, high-carbohydrate (HFHC) diet for 36 weeks. In this diet, carbohydrates were derived mainly from maize starch and sucrose, proteins from casein and soybean meal, and fats from a mixture of lard and soybean oil. The HFHC diet therefore provided a higher proportion of simple sugars and saturated fat compared to the control diet. All animals had free access to their respective diets and water ad libitum. No deaths were observed during the feeding period.

### 2.4. Mating

First, 40-week-old female Sprague-Dawley rats (n = 18) were mated with males of the same strain. Briefly, two females were housed in one cage to allow their estrus cycles to become synchronized. The ovarian cycle of rats was between four and five days long. Daily vaginal smears were taken, and when the female rats were in the pro-estrus stage of their cycle, a male rat was introduced into the cage. The following day, vaginal smears were taken from the female rats, and the presence of sperm indicated successful mating, and cytology was used to confirm positive pregnancy. The day of fertilization was regarded as GND 0 [13]. The male rats were then removed from the cage and sacrificed after successful mating.

### 2.5. Induction of PE

The pregnant dams (n = 6) were given 0.3 g/l a day of N*^ω^*-nitro-l-arginine methyl ester (L-NAME) in drinking water from GND8 to GND 14 [14]. Blood pressure and proteinuria were determined to confirm the successful induction of PE on GND 9. Blood pressure was monitored using a non-invasive tail cuff method with photoelectric sensors (IITC Model 31 Computerised Blood Pressure Monitor, Life Sciences, Woodland Hills, CA, USA). The animals warmed at ±30 °C in an enclosed chamber (IITC Model 31 Computerised Blood Pressure Monitor, Life Sciences, Wood land Hills, CA, USA) for 30 min before taking blood pressure readings.

### 2.6. Plasma and Tissue Collection

On GND 19, pregnant rats were sacrificed by decapitation for the collection of the liver tissue, whole blood, and plasma. Plasma was obtained by collecting blood in single precooled heparinized containers, and the blood was centrifuged at 1000× *g* for 10 min in a Hermle Laborechnic GmBH centrifuge (Wehingen, Germany). The liver tissues were removed, weighed, and then snap-frozen in liquid nitrogen. Afterwards, the liver tissues and plasma were stored in an Ultra Bio Freezer (Snijers Scientific, Tilburg, The Netherlands) at −80 °C for biochemical tests.

The relative liver weight of all the animals in each experimental group was determined from the percentage of the ratio of liver weight to body weight.

relative liver weight = (liver weight × 100)/body weight

### 2.7. Biochemical Tests

#### 2.7.1. Lipid Profile

The preparation of liver tissue samples and the homogenate medium used to determine hepatic triglyceride was conducted according to the manufacturer’s instructions in the triglyceride assay kit (Catalog No: E-BC-K238; Manufacturer: Elabscience, Houston, TX, USA). Briefly, liver tissue was harvested; then tissue was washed with cold PBS (0.01 M, pH 7.4). A total of 20 mg of liver tissue was homogenized in 180 µL homogenate medium with a Dounce homogenizer (Merck Life Science (Pty) Ltd., Johannesburg, South Africa) at 4 °C. The sample was centrifuged at 12,000× *g* for 10 min at 4 °C to remove insoluble material. The supernatant was collected and kept on ice for detection, and the protein concentration of the supernatant was determined using BCA Protein Colorimetric Assay Kit (E-BC-K318-M). A total of 2.5 µL of distilled water was added to the blank well, 2.5 µL of the standard was added to the standard well, 2.5 µL of the sample was added in each sample well, and 250 µL of a working solution was added in all the wells and mixed thoroughly. The plate was incubated at 37 °C for 10 min, and the OD value was measured at 510 nm with a microplate reader.

#### 2.7.2. Antioxidant Profile

The antioxidant profile of the liver was determined by measuring the concertation of SOD 1 according to the manufacturer’s instructions (Catalog No: E-EL-R1424; Elabscience Biotechnology Co., Ltd., Houston, TX, USA) via the microplate reader, SPECTROstar Nano spectrophotometer (BMG LABTECH, Ortenburg, LGBW, Germany). Briefly, for the SOD 1 ELISA kits, 50 µL of each dilution of the standard, blank, and sample was added into the appropriate wells; thereafter, 50 µL of biotinylated detection Ab working solution was added to each well immediately. The plate was covered with a sealer and incubated for 45 min at 37 °C. The plate was washed 3× with 350 µL of buffer in each well. After the washing step, 100 µL of HRP conjugate working solution was added to each well, and the plate was incubated for 30 min at 37 °C. After incubation, the solution in each well was decanted, and this process was repeated 5×. A total of 90 µL of substrate reagent was added to each well, and the plate was incubated for 15 min at 37 °C. Subsequently, 50 µL of the stop solution was added, and the optical density (OD) of each well was determined using a microplate reader at 450 nm.

#### 2.7.3. Lipid Peroxidation

The MDA concentration in the liver was determined to estimate the amount of lipid peroxidation according to a previously described protocol by [15]. Briefly, liver tissue (50 mg) was homogenized in 500 μL of 0.2% phosphoric acid. The homogenate was centrifuged for 10 min at 400× *g*. Thereafter, 400 μL of the homogenate was supplemented with 400 µL of 2% phosphoric acid. The solution was aliquoted into two separate tubes. Then, 200 μL of 7% phosphoric acid was added into both glass tubes while adding 400 μL of 3 mM hydrochloric acid (HCl) into one glass test tube (blank) and 400 μL thiobarbituric acid (TBA)/butylated hydroxytoluene (BHT) into the other test tube (sample test). To achieve an acidic pH of 1.5, 200 μL of 1M HCl was added to both the blank and sample test tubes. Both solutions were heated for 15 min at 100 °C and allowed to cool to room temperature. After, 1.5 mL butanol was added to the cooled solution; the sample was vortexed for 1 min to achieve rigorous mixing and allowed to settle until two phases could be observed. The butanol phase/top layer was transferred to Eppendorf tubes and centrifuged for 6 min at 13,200× *g*. The samples were aliquoted into a 96-well microtiter plate in triplicate, and a Biotek mQuant spectrophotometer (Biotek, Johannesburg, South Africa) was used to determine the absorbance at a 532 nm reading.

#### 2.7.4. Liver Inflammatory Markers

For IL 6 and TNF α measurements, 50 mg of liver tissue was homogenized, and the concentration was determined using an ELISA kit following the manufacturer’s instructions (catalog no: E-EL-R0015 and catalog no.: E-EL-R2856; manufacturer: Elabscience Houston, TX, USA). IL6 and TNF α ELISA kits were used. Briefly, 100 µL standard or sample was added to each well, and the plate was incubated for 90 min at 37 °C. After incubation, the liquid was removed, and 100 µL of biotinylated detection Ab was added and incubated for 1 h at 37 °C; the plate was washed 3×. A total of 100 µL of HRP conjugate was added and incubated for 30 min at 37 °C. The plate was washed 5×; then 90 µL of substrate reagent was incubated for 15 min at 37 °C, and 50 µL of stop solution was added, and the plate was immediately read at 450 nm. Protein detection was performed as follows: Liver tissue was harvested; then tissue was washed with cold PBS (0.01 M, pH 7.4). A total of 20 mg of liver tissue was homogenized in 180 µL homogenate medium with a Dounce homogenizer at 4 °C. The sample was centrifuged at 12,000× *g* for 10 min at 4 °C to remove insoluble material. The supernatant was collected and kept on ice for detection, and the protein concentration of the supernatant was determined using BCA Protein Colorimetric Assay Kit (E-BC-K318-M).

#### 2.7.5. Liver Enzymes

Liver damage biomarkers, including alanine aminotransferase (ALT), aspartate aminotransferase (AST), total bilirubin (TBIL), and albumin, were measured in plasma using the Catalyst One Chemistry Analyzer (IDEXX Laboratories, Westbrook, ME, USA).

#### 2.7.6. Hematological Analysis

Red blood cell (RBC) count, hemoglobin (Hb), and platelet concentration were measured in the blood collected from all the rat groups using a hematocytometer (Beckman Coulter, Indianapolis, IN, USA).

### 2.8. Statistical Analysis

The sample size (n) for this study was calculated using G*Power (version 3.1.9.7), a statistical power analysis software. This was performed to ensure that the sample size was adequate to detect statistically significant differences between the groups while maintaining ethical considerations for animal use. Statistical analysis was conducted using GraphPad Prism Software (version 9.00, GraphPad Software, San Diego, CA, USA). A normality test (Shapiro–Wilk test) was performed followed by a parametric one-way ANOVA. To analyze differences between the controls and the experimental group, Bonferroni’s multiple comparisons test was performed. Values of *p* < 0.05 were used to indicate statistical significance.

## 3. Results

### 3.1. Liver Weight and Relative Liver Weights

**Figure 2:**
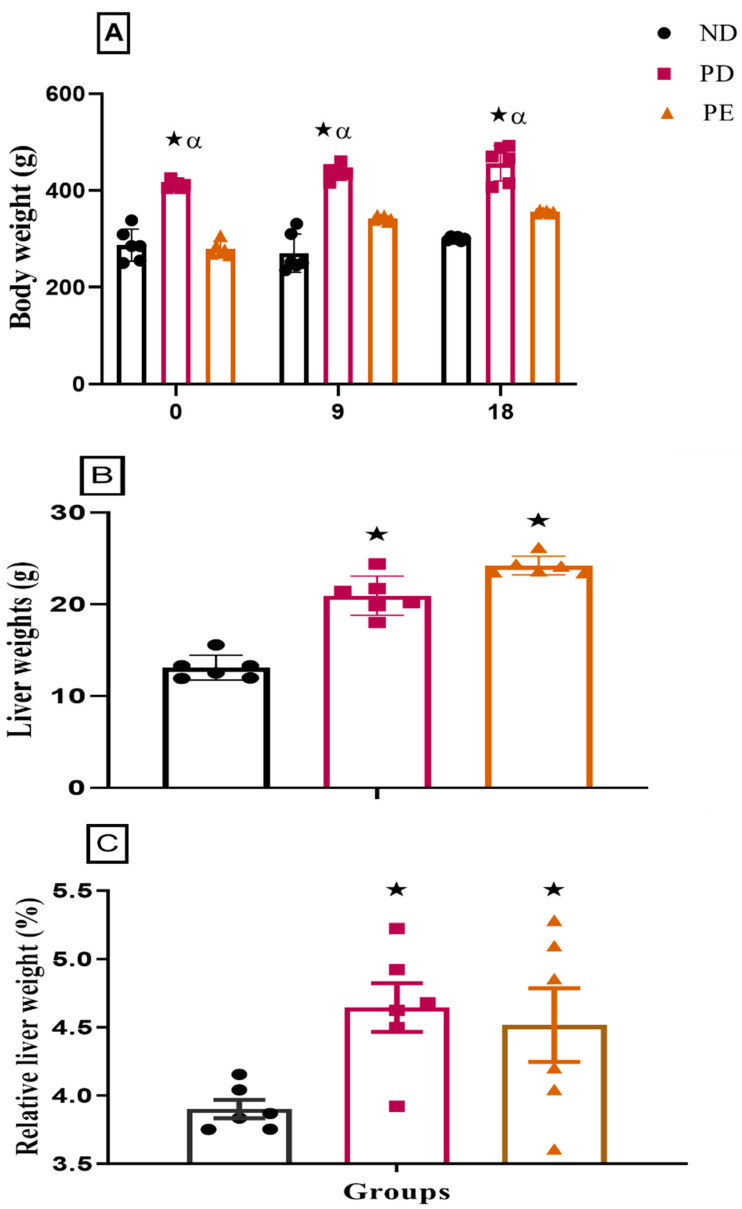
(**A**) Body weights, (**B**) liver weights, and (**C**) relative liver weights of normal pregnant rats and pregnant prediabetic and pregnant preeclamptic rats measured after 19 days of gestation. Values are presented as means and SD (n = 6 in each group). 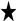 *p* ˂ 0.05 by comparison with normal pregnant group. α *p* < 0 05 by comparison to pregnant preeclamptic group.

Both the preeclamptic pregnant and prediabetic groups showed a significant increase in both liver weights and relative liver weights when compared to the normal pregnant control group (*p* < 0.05; Figure 2). The prediabetic pregnant group presented with significantly increased body weight throughout gestation when compared to the normal pregnant control and preeclamptic pregnant group (*p* < 0.05; Figure 2). The preeclamptic pregnant group showed a consistent body weight throughout gestation when compared to the normal pregnant control group (*p* < 0.05; Figure 2).

### 3.2. Non-Fasting Blood Glucose

**Figure 3:**
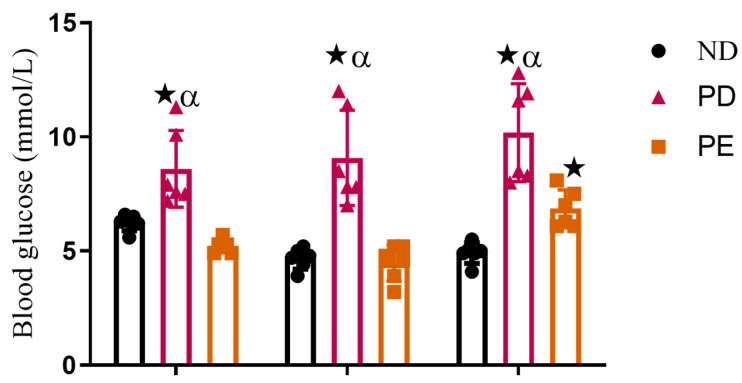
Non-fasting blood glucose and triglyceride concentrations of normal pregnant rats, pregnant prediabetic rats, and pregnant preeclamptic rats over period of 18 gestational days. 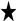 *p* ˂ 0.05 by comparison with normal pregnant group. α *p* < 0 05 by comparison to pregnant preeclamptic group.

The prediabetic pregnant group presented with significantly higher glucose concentrations throughout gestation when compared to the normal pregnant control group (*p* < 0.05; Figure 3). The preeclamptic pregnant group showed significantly increased blood glucose concentrations on GND18 when compared to the normal pregnant control group (*p* < 0.05; Figure 3) over the experimental period.

### 3.3. Liver Triglyceride (TG) Concentration

**Figure 4:**
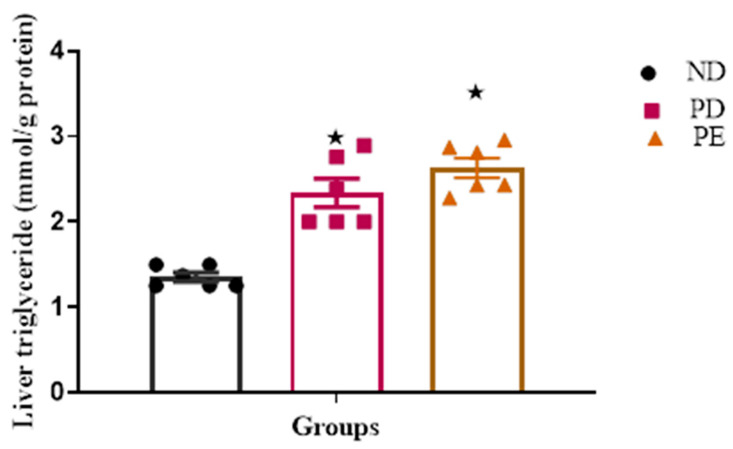
Liver TG concentrations tested after 19 days of gestation. Values are presented as means and SD (n = 6 in each group). 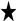 *p* ˂ 0.05 by comparison with normal pregnant group.

Both the prediabetic pregnant and preeclamptic groups showed significantly increased liver TG concentrations when compared to the normal pregnant control group (*p* < 0.05; Figure 4).

### 3.4. Oxidative Stress Marker

The pregnant preeclamptic group showed significantly increased MDA concentrations and a decreased activity of SOD in comparison with the normal pregnant group (*p* ˂ 0.05; Table 2). The pregnant prediabetic group showed significantly increased MDA concentrations and a decreased activity of SOD in comparison with the normal pregnant group, which were similar results to those for the pregnant preeclamptic group (Table 2).

### 3.5. Inflammatory Markers (IL-6 and TNFα)

**Figure 5:**
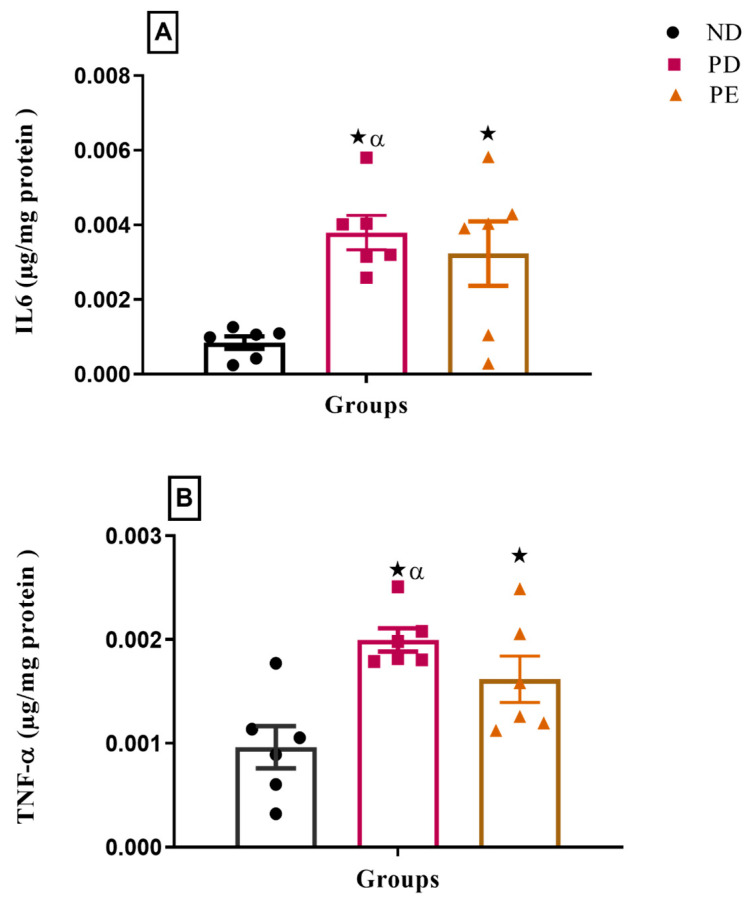
Inflammatory markers (**A**) IL6 and (**B**) TNFα on the liver of normal pregnant rats, pregnant prediabetic and pregnant preeclamptic, measured after 19 gestational days. The values are presented as means and SD (n = 6 in each group). 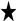 *p* ˂ 0.05 by comparison with the normal pregnant group. α *p* < 0.05 by comparison to the pregnant preeclamptic group.

The results showed that both the plasma IL-6 and TNFα concentrations of the preeclamptic pregnant and prediabetic groups were significantly higher than those of the normal pregnant control (*p* < 0.05; Figure 5).

### 3.6. Plasma Liver Enzymes: Albumin and Bilirubin Concentrations

**Figure 6:**
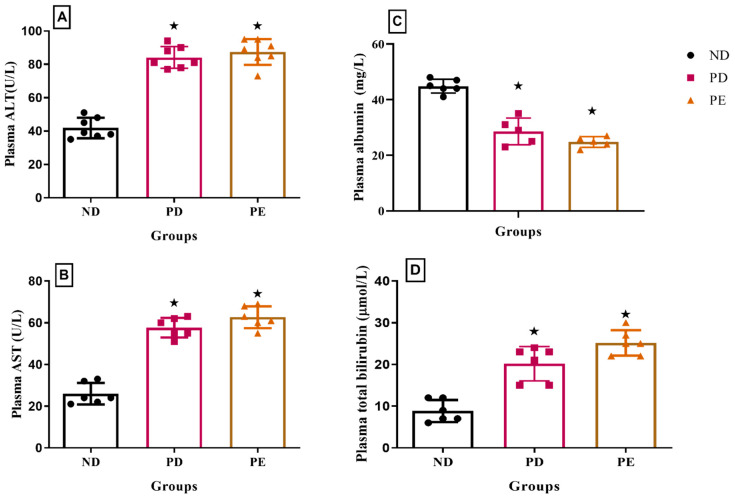
(**A**) Plasma ALT, (**B**) AST, (**C**) albumin, and (**D**) bilirubin concentrations of normal pregnant rats and pregnant prediabetic and pregnant preeclamptic rats measured after 19 gestational days. Values are presented as means and SD (n = 6 in each group). 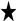 *p* ˂ 0.05 by comparison with normal pregnant group.

The results showed that both the plasma AST and ALT concentrations of the pregnant prediabetic group and pregnant preeclamptic group were significantly higher than those of the normal pregnant control (*p* < 0.05; Figure 6). The concentration of plasma bilirubin was significantly increased in both the pregnant prediabetic group and pregnant preeclamptic group when compared to the normal pregnant control (*p* < 0.05; Figure 6). Both the prediabetic pregnant and preeclamptic groups showed significantly decreased plasma albumin concentrations when compared to the normal pregnant control group (*p* < 0.05; Figure 6).

### 3.7. Hematological Parameters

**Figure 7:**
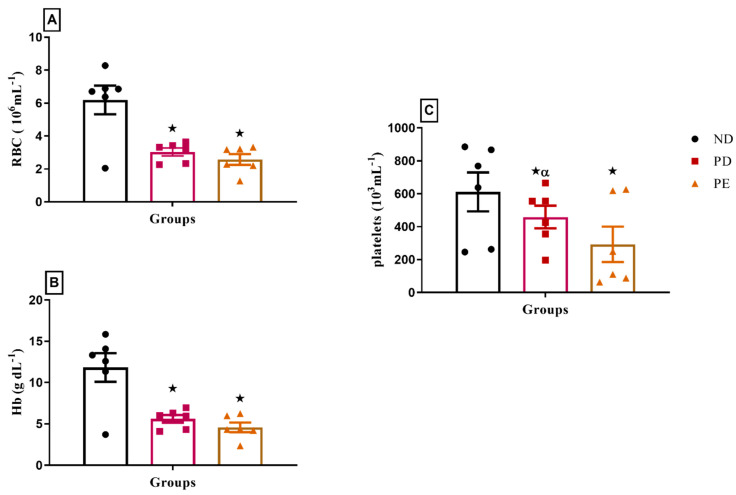
(**A**) RBC, (**B**) Hb, and (**C**) platelet concentrations of normal pregnant rats and pregnant prediabetic and pregnant preeclamptic rats measured after 19 gestational days. Values are presented as means and SD (n = 6 in each group). 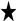 *p* ˂ 0.05 by comparison with normal pregnant group. α *p* < 0.05 by comparison to pregnant preeclamptic group.

Both pregnant prediabetic and pregnant preeclamptic rats exhibited significant decreases in hematological parameters, including red blood cell count (RBC), hemoglobin (Hb), and platelet concentration, compared to normal pregnant rats (*p* < 0.05; Figure 7).

## 4. Discussion

The results of the present study showed that pregestational PD is a risk factor for HELLP syndrome, with the observation that metabolic complications originating at the onset of prediabetes were associated with liver dysfunction, inflammatory activation, and hematological alterations during pregnancy. Specifically, the PD pregnant group exhibited increased liver weights and triglyceride levels, elevated plasma liver enzyme levels, liver inflammation, reduced red blood cell counts and hemoglobin, and decreased platelet counts, given that these alterations resemble the hepatic and hematological disturbances observed in HELLP syndrome. The diagnosis of HELLP syndrome requires the presence of microangiopathic hemolysis, thrombocytopenia, and abnormalities of liver enzymes [16]. Pregnant women who do not have all these parameters are considered to have partial HELLP syndrome [17]. The aim of this study was to investigate pregestational PD as a risk factor for HELLP syndrome, which is one of the complications of severe PE [17].

There is a direct relationship between maternal TGs and newborn weight [18]. This has been shown in both animal and human studies [18,19,20]. Lipids have been shown to play a major role in fetal development; however deviations in maternal hyperlipidemia trigger pathogenic events such as atherosclerosis and cardiovascular disease (CVD) later in a child’s life [21]. In this study, we observed an increase in TG concentration in both the pregnant PE and pregnant PD groups in comparison to the normal pregnant control. The symptoms of PE are caused by endothelial dysfunction related to hypoxia in the placenta and abnormal lipid metabolism [9]. Lipid abnormalities in PE have been shown to be associated with liver steatosis [22]. PD is associated with insulin resistance, which can increase peripheral lipolysis, triglyceride synthesis, and hepatic uptake of free fatty acids, which ultimately leads to nonalcoholic fatty liver disease (NAFLD) [23]. NAFLD has been shown to have adverse effects on pregnancy-related outcomes and maternal and child health [24]. These adverse effects include hypertensive complications of pregnancy, PE, preterm birth, and low birth weight [25].

The accumulation of TGs in the liver of the pregnant PE and pregnant PD groups is associated with the increased relative liver weights in both groups and the development of oxidative stress and reduced antioxidant activity in the liver in both the pregnant PE and PD groups in comparison to the normal pregnant group. In line with this, our results demonstrated reduced SOD levels and increased MDA levels, confirming the presence of oxidative stress and lipid peroxidation in these groups. Oxidative stress causes hepatocellular damage through several different mechanisms, including lipid peroxidation, that can directly stimulate cell necrosis and the activation of apoptosis [26]. Hepatic fat accumulation and oxidative stress can cause an inflammatory response followed by necrosis, fibrosis, and cirrhosis, ultimately leading to liver failure [27,28]. We observed increased inflammatory marker levels (TNF and IL 6) in both the pregnant PE and PD groups in comparison to the normal pregnant group.

These inflammatory mediators have been shown to activate stellate cells and scar tissue deposition in the liver [29]. Hepatocyte inflammation and hepatic fat accumulation are associated with hepatocyte injuries, which lead to the release of transaminase enzymes such as ALT and AST [30,31]. In this study, the levels of these enzymes were significantly elevated in the pregnant PE and pregnant PD groups in comparison with the normal pregnant group. The liver enzyme levels in plasma reflect the presence of injury in the liver as these enzymes are components of hepatocytes that are released into the circulation upon hepatocyte damage. The serum levels of liver enzymes can also be used as a marker of hepatic fat deposition [32,33]. The decrease in plasma albumin observed in both PD and PE in comparison with the normal pregnant group is associated with impaired liver synthetic function.

The stressed placenta in HELLP releases antiangiogenic factors, ROS (oxidative stress) which interact with vascular endothelial cells (ECs) and induce the release of inflammatory cytokines in the maternal circulation [34]. The damaged vascular endothelium leads to changes in RBCs as the RBCs pass through the vessels, which results in microangiopathic hemolytic anemia [34]. The pregnant PE group presented with significantly reduced platelet count, which correlated with a low RBC count and low hemoglobin concentration in comparison to the normal pregnant group. Hepatocyte damage in HELLP is enhanced by microangiopathy, which impedes portal blood flow [35]. In PE, the ratio of the prostacyclin–thromboxane production rate is decreased, favoring the vasoconstrictive thromboxane, and both these substances also play a role in the event of thrombocytopenia [36].

Hyperglycemia induces oxidative damage which is associated with the reduced function of red blood cells (RBCs) due to reduced levels of NADPH, which is necessary for antioxidant enzymatic activity [37]. T2DM increases RBC concentration, causing an increase in blood viscosity and the development of high blood pressure [37]. IL6 is elevated in T2DM and has been shown to promote the apoptosis of immature erythrocytes, thus causing a reduced number of circulating RBCs and in turn causing a reduction in circulating hemoglobin [38]. The pregnant PD group had significantly low RBC and low hemoglobin levels in comparison to the normal pregnant group. RBCs in T2DM are stiffened by chronic hyperglycemia, causing them to have a harmful effect on the vasculature [39]. These stiff RBCs have been shown to damage the endothelium, in addition to the damage caused by oxidative stress [39]. These deformed RBCs are therefore susceptible to degradation by Kupfer cells, which could partly explain increases in bilirubin in this study. Additionally, the change in the composition and function of RBCs in T2DM causes endothelium aggregation. This is also considered an effect that facilitates the adhesion of platelets, which results in thrombus formation in T2DM. A study in prediabetic male Sprague-Dawley rats showed that RBC abnormalities are not enough to cause thrombus formation [40]. In the prediabetic state, there is a progressive deterioration in the structural properties of RBCs [40].

Although PD and HELLP syndrome can both cause liver dysfunction, in this study, the rats were treated separately for PD and PE, so any liver effects observed cannot be attributed to a shared mechanism between the two conditions. Therefore, further studies are needed to explore potential links and underlying mechanisms.

## 5. Conclusions

The findings of this study indicate that pregestational PD contributes to changes in liver function and hematological parameters that resemble aspects of HELLP syndrome. The PD pregnant group exhibited increased liver weights, elevated liver triglyceride levels, elevated plasma liver enzyme levels, liver inflammation, decreased red blood cell counts and hemoglobin, and decreased platelet counts, suggesting that pregestational PD may predispose patients to HELLP-like complications.

These results highlight the potential importance of the early identification and management of pregestational PD to reduce the risk of severe hypertensive pregnancy disorders.

## Figures and Tables

**Table 1:** Composition of ingredients in high-fat, high-carbohydrate diet.

Nutrient	Units	Actual	Nutrient	Units	Actual
Dry Matter	g/kg	919.93	Available Total Sulfur Amino Acids (ASTSAA)	g/kg	6.79
Metabolizable Energy	MJ/kg	15.86	Asvaline	g/kg	5.80
Crude Protein	g/kg	151.27	Fat	g/kg	250.46
Threonine	g/kg	4.51	Carbohydrate	g/kg	427.29
Isoleucine	g/kg	5.24	Fiber	g/kg	22.08
Lysine	g/kg	6.54	Ash	g/kg	26.31
Methionine	g/kg	4.86	Available Phosphorus (Avl-P)	g/kg	1.66
Tryptophan	g/kg	1.30	Calcium	g/kg	5.47
Astatine	g/kg	3.30	Total Phosphorus	g/kg	3.60

**Table 2:** The effects of PE and pregestational PD on MDA concentrations and SOD activity in the liver tissue over 19 days of gestation. Values are represented as means and SD (n = 6 in each group).

Groups	MDA (nmol/g Protein)	SOD (pg/mg Protein)
ND	0.025 ± 0.001	2.077 ± 0.172
PD	0.053 ± 0.005 *α	1.134 ± 0.231 *α
PE	0.049 ± 0.004 *	0.528 ± 0.144 *

* *p* ˂ 0.05 by comparison with normal pregnant group. α *p* ˂ 0.05 by comparison with preeclamptic pregnant group.

## Data Availability

All data supporting the findings of this study are available within the manuscript. No additional datasets were generated or analyzed.
